# Pharmacological Interventions for the Treatment and Control of Shivering in Adult Patients Undergoing Elective Surgery Under Regional Anaesthesia: A Systematic Review and Meta-Analysis

**DOI:** 10.5152/TJAR.2021.20008

**Published:** 2022-08-01

**Authors:** Malika Hameed, Naureen Akber Ali, Khalid Ahsan, Mohsin Nazir

**Affiliations:** 1 Department of Anaesthesiology, Aga Khan University Hospital, Karachi, Pakistan; 2 Department of Nursing and Midwifery, Aga Khan University Hospital, Karachi, Pakistan

**Keywords:** Adverse effects, central neuraxial block, intraoperative shivering, pharmacologic interventions, regional block

## Abstract

Intraoperative shivering is quite common after regional anaesthesia, which not only increases the total body oxygen requirement but also causes discomfort to the patients. The aim of this systematic review is to determine the effectiveness of pharmacological agents administered intra-operatively for treating shivering in adult patients who are undergoing elective surgery under regional (i.e., central neuraxial) anaesthesia so that an optimal choice of an agent can be recommended for clinical application. A literature search was carried out using PubMed, Cochrane Library, CINAHL databases, and hand searches to identify relevant studies. After literature screening and information extraction, a systematic review was performed. Meta-analysis was performed for the primary outcome. The primary outcome was to evaluate the effectiveness of pharmacological agents used for the treatment and control of intraoperative shivering and the time taken to control shivering. The secondary outcome includes recurrence of shivering after pharmacological intervention and identification of common adverse effects related to them. In total, 10 studies (791 patients) were included. Common interventions were opioids, central α2 receptor agonist, and few other medications like magnesium sulfate, ondansetron, nefopam, and amitriptyline. Tramadol and dexmedetomidine were the most frequently documented drugs compared with other drugs to resolve shivering. The most effective drug with approximately 100% response rate was dexmedetomidine with the dose of 0.5 μg kg^−1^ intravenously given just after the appearance of shivering. Studies showed that tramadol is also an effective drug used to control shivering in most patients, and its effect is comparable with the pethidine.

**Main Points**

- Shivering occurring during regional anaesthesia is quite common. It not only has detrimental effects, but it also causes discomfort to the patient during surgery.
- This systematic review evaluates the effectiveness of pharmacological agents administered intra-operatively for treating shivering in adult patients undergoing elective surgery under regional (i.e., central neuraxial) anaesthesia so that an optimal choice of an agent can be recommended for clinical application.
- The most effective drug with approximately 100% response rate was dexmedetomidine with the dose of 0.5 μg kg^−1^ intravenously given just after the appearance of shivering.
- Studies showed that tramadol is also an effective drug used for the control of shivering in most patients and its effect is comparable with the pethidine.

## Introduction

Shivering is defined as an involuntary rhythmic activity of skeletal muscles in response to hypothermia. Shivering occurring during regional anaesthesia is quite common. It is due to loss of thermoregulatory vasoconstriction, which prevents redistribution of heat, leading to an increase in oxygen consumption, cardiac workload, an increase in carbon dioxide production, and an increase in metabolic rate.^1^ It not only has detrimental effects, but it also causes discomfort to the patient during surgery. Hence it is important to know measures to control this complication.

The primary objective of this systematic review was to determine the effectiveness of various pharmacological agents administered intra-operatively after the development of shivering to treat and control it in adult patients undergoing elective surgery under regional anaesthesia. Our secondary objectives were to identify any adverse effects related to the administration of these agents.

## Methods

This systematic review was performed in accordance with the Preferred Reporting Items for Systematic Reviews and Meta-analyses (PRISMA) statement.^2^ The protocol was registered in PROSPERO (www.crd.york.ac.uk/PROSPERO) with the unique identification number CRD42018117713.

A systematic search was performed from PubMed, Cochrane Controlled Trials Register, and CINAHL databases for articles published in any year inclusive using the search items (including alternative spelling) for pharmacological interventions, intraoperative shivering, regional anaesthesia, spinal anaesthesia, epidural anaesthesia, combined spinal-epidural anaesthesia, and neuraxial anaesthesia. The search was restricted to publish English language articles only.

Additional studies were identified through the reference list of relevant articles. Authors of identified publications were not contacted for additional information. Search strategy with relevant search terms is included in a separate supplemental content file.

### Study Selection Criteria

Inclusion criteria for considering studies for this systematic review were as follows:

Only prospective randomized controlled trials (RCT) where the title or the objective of the study mentioned the effect of pharmacological agents on treatment and control of intraoperative shivering under regional anaesthesia were included. Both blinded and unblinded studies were included.

Studies including adult patients of any race and either gender undergoing elective surgery under regional anaesthesia in the operating room were reviewed.

Studies in which specific pharmacological agents were used through any route for the treatment and control of shivering occurring intra-operatively were included.

Studies that investigated one or more of the following outcome measures related to shivering response were included.

Treatment and control of intraoperative shivering and time taken by the drug to treat shivering were primary outcome measures. Recurrence of shivering after pharmacological intervention and any adverse effects of the given drugs were secondary outcome measures.

The following studies were excluded:

Studies that investigated the effect of pharmacological intervention in the pediatric population and crossover studies were excluded. We also excluded studies where pharmacological intervention was done before regional anaesthesia was given.

The data were extracted individually by the 3 independent reviewers who documented information on participants, methods, interventions, outcomes, and bias assessment. The reasons for inclusion or exclusion of studies were also noted. Any disagreement was referred to the senior reviewer.

The risk of bias assessment was done by 3 reviewers according to standard descriptions for each type of bias as per definitions given in the Cochrane Handbook for Systematic Reviews of Intervention.^3^ Random sequence generation, allocation concealment, blinding of participants, blinding of outcome assessment, the bias of incomplete outcome data, and selective reporting bias were assessed.

All the included trials were not comparing the treatment drug with placebo, as mentioned in the registered protocol (PROSPERO). We only found 3 trials^4-6^ that were placebo-controlled. The rest of the trials consisted of a comparison of 2 or more than 2 treatment drugs.

### Statistical Analysis

Review Manager 5.3 software was used for meta-analysis. Two or more than 2 studies with similar comparisons and outcomes were selected for meta-analysis. Meta-analyses of 2 studies with tramadol versus pethidine for the control of intraoperative shivering^7,8^ and three studies with dexmedetomidine versus tramadol for the time to control shivering^8-10^ were conducted. The pooled estimates were obtained through a random effect model with Mantel-Haenzel weighting and the amount of heterogeneity among pooled studies was quantified through the Chi^2^ test and I^2^ index. The pooled effect estimates for shivering control were measured in odds ratio (OR), and time to control shivering was measured through mean difference (MD) with 95% confidence intervals. Standard normal statistic (Z-score) was used to estimate the *P* value of the null hypotheses that (i) there was no difference in the effect of tramadol and pethidine in shivering control and (ii) there was no difference in the time to control shivering between dexmedetomidine and tramadol. A significance level of *P* < .05 was considered statistically significant.

## Results

**Supplementary Figure 1.**
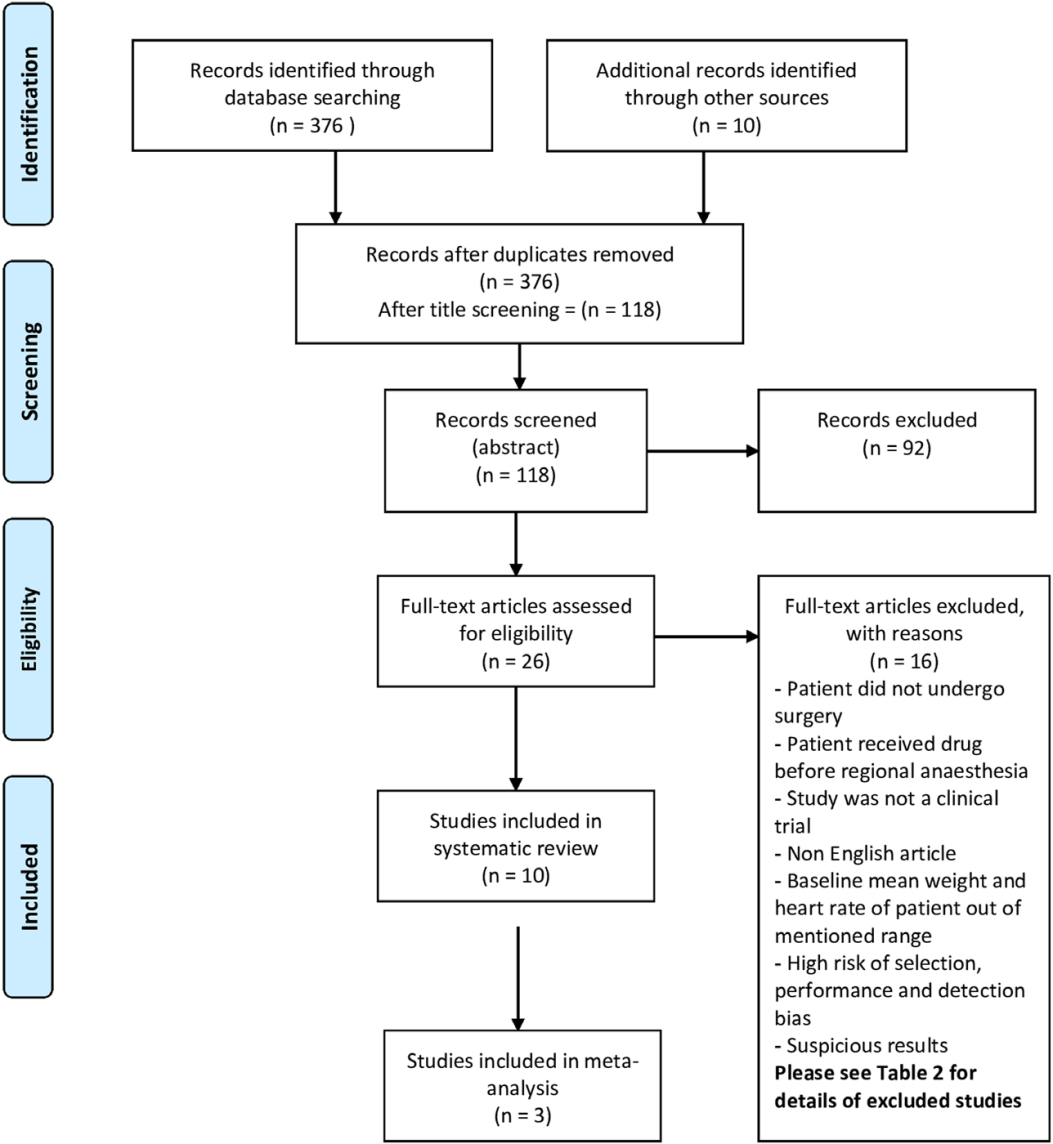
PRISMA flow diagram.

We identified 26 studies; however, only 10 studies met our eligibility criteria. The literature search and study selection are shown in the PRISMA flow diagram (Supplementary Figure 1). Details of included studies were summarized in Supplementary Table 1. All studies that were included in the literature review were published from 1988 to 2018.

All included studies were evaluated for their quality (Supplementary Table 1). A total of 791 adult participants, both male and female greater than 18 years of age, who were undergoing elective surgery were included from the selected studies. Drugs were given by the intravenous (IV) route to all those participants. Four studies compared 2 groups,^7,10-12^ while 6 studies compared 3 groups using standard alternatives as control.^4-6,8,9,13[Bibr b10-tjar-50-4-246]^ A saline control group was used in 3 studies.^4-6^

Sixteen full-text studies were excluded after the full review. Details and reasons for exclusion have been summarized in Supplementary Table 2.

Tramadol and dexmedetomidine were the most frequently documented drugs compared with other drugs to resolve shivering. Out of 10 studies, tramadol was studied in 6 studies^4,7-9, 11, 13^ and dexmedetomidine was also used in 6 studies.^6,8,9,10-12^ Clonidine was used in 3 studies^9,12,13^ and meperidine was used in 2 studies.^7,8^ Other drugs used were nalbuphine,^6^ magnesium sulfate,^5^ nefopam,^11^ and butorphanol.^13^ Saline was used as a control in 3 studies.^4-6^

### Studied Outcome

1. Control of shivering
2. Time to control shivering
3. Recurrence of shivering
4. Adverse effects
5. Meta-analysis

### Control of Shivering

Control of shivering was reported in 7 out of 10 included trials,^4,6,7,8,11- 13^ but the method of reporting was inconsistent. A study by Bansal and Jain^10^ reported shivering control as complete or incomplete. Chan et al^4^ reported it in graphical form with categories of no improvement, slight improvement, and marked improvement. Another study measured it as patients’ assessed treatment efficacy in categories of no improvement, partial improvement, and marked improvement.^8^ A study by Kaya et al^7^ reported the efficacy of treatment for shivering in graphical form and in percentages and presented results in terms of improvement (Grades 0, 1, and 2) and no improvement (Grades 3 and 4). One study by Megalla and Mansour^6^ reported it as a success rate in percentages. Two studies measured control of shivering in the form of response rate percentage (%) and numbers (n).^11,12^

Fern and Misiran reported the comparison of dexmedetomidine 0.5 μg kg^−1^, pethidine 0.5 mg kg^−1^, and tramadol 0.5 mg kg^−1^ in 20 patients per group. The treatment efficacy was 100% in the dexmedetomidine group, 85% in the pethidine group, and 55% in the tramadol group. The response rate in the dexmedetomidine group was only significant when compared to the tramadol group (*P* = .0012). It was also observed that although the response rate was higher in the pethidine group than in the tramadol group, the difference was not statistically significant (*P* = .082).^8^

Megalla and Mansour^6^ compared nalbuphine 0.07 mg kg^−1^ (Group N) and dexmedetomidine 0.5 μg kg^−1^ (Group D) with saline (Group C) in 25 patients per group for treatment of shivering. The success rate was 100% in group D, 92% in Group N, and only 32% in C (saline) group. A statistically significant difference (*P* < .0001) in response rate was observed when Group N and Group D were compared to Group C.

Mohamed^11^ compared the dexmedetomidine 0.5 μg kg^−1^ (Group D) and Nefopam 0.15 mg kg^−1^ (Group N) in 50 patients per group. The response rate of the disappearance of shivering was 96% in Group D and 100% in Group N. There was no statistically significant difference in the response rate of dexmedetomidine and nefopam (*P* = .492).

Panneer et al^12^ compared 2 groups of 30 adult patients each, who received intravenous clonidine (Group C; 1 μg kg^−1^) and dexmedetomidine (Group D; 0.5 μg kg^−1^). Dexmedetomidine group showed a statistically significant response rate (100% vs 80%) when compared to the clonidine group (*P* < .05).

Kundra et al^9^ studied the effect of intravenous dexmedetomidine (0.5 μg kg^−1^) with tramadol (0.5 mg kg^−1^) in 100 patients to control shivering. Cessation of shivering occurred in all patients in both groups.

Bansal and Jain^10^ reported a comparison of tramadol 50 mg IV bolus, butorphanol 1 mg IV bolus, and clonidine 150 μg intravenous bolus in 30 patients per group for shivering control. The rate of complete shivering control was 73.3% in tramadol, 83.3% in butorphanol, and 53.3% in clonidine groups. There was a significant response rate when clonidine was compared with butorphanol (*P* = .012); however, the comparison of clonidine with tramadol (*P* = .10) and butorphanol with tramadol (*P* = .34) was statistically insignificant.

Chan et al^4^ compared 2 doses of intravenous tramadol (T0.5 = 0.5 mg kg^−1^ and T0.25= 0.25 mg kg^−1^) with saline placebo 0.05 mL kg^−1^ in 36 patients. Shivering was controlled in 92% (n = 12) of patients in Group T0.25, 83% (n = 10) in Group T0.5, and 27% (n = 3) in group saline. The response rate of group T0.5 and group T0.25 was statistically insignificant. However, both groups were statistically significant when compared with the saline group (*P* <.001, Kruskal-Wallis ANOVA).

Kaya et al^7^ compared meperidine 0.35 mg kg^−1^ to tramadol 0.25 mg kg^−1^ (n = 30). The effect on shivering control was quite similar in both drugs: meperidine (93%) and tramadol (90%) (*P*  = .67 using a 2 sample test for proportion, z test in STATA). However, the study summarized that tramadol might be a suitable alternative to the meperidine in patients experiencing intraoperative shivering.

Ibrahim et al^5^ did a double-blinded placebo-controlled trial on 120 patients. In Group P (prophylactic; n = 40) MgSO_4_, 50 mg kg^−1^ intravenous bolus followed by 2 mg kg^−1^ h^−1^ infusion was administered. In Group T (therapeutic; n = 40), MgSO_4_ 50 mg kg^−1^ intravenous bolus only was given as therapy when shivering began. In Group C (control), saline was given at identical times. Grade 3 shivering was significant with reported frequencies of 15%, 45%, and 50% in Group P, Group T, and Group C, respectively. Therapeutic MgSO_4_ was more effective for control of shivering with no utilization of rescue meperidine as compared to Group P (20% use of rescue meperidine, *P*  < .05) and Group C (50% use of rescue meperidine, *P*  < .01).

### Time Taken to Control Shivering

Out of 10 studies, 9 studies reported the (mean ± SD) time taken to resolve shivering.^4,6-13^

Fern and Misiran^8^ reported the comparison of dexmedetomidine 0.5 μg kg^−1^, pethidine 0.5 mg kg^−1^, and tramadol 0.5 mg kg^−1^ in 30 patients per group. He reported that tramadol takes less time to abort shivering than meperidine and dexmedetomidine. However, the time to control shivering with the use of tramadol (5.9 ± 2.1 minutes) was statistically insignificant when compared to the dexmedetomidine (7.3 ± 3.8 minutes, *P*   = 0.15 using two-sample *t*-test in STATA software) and pethidine (6.2 ± 2.3 minutes, *P*  = .66 using two-sample *t*-test in STATA software).

Kundra et al^9^ studied the effect of intravenous dexmedetomidine (0.5 μg kg^−1^) with tramadol (0.5 mg kg^−1^) and reported that the time taken for the cessation of shivering was significantly less with dexmedetomidine (2.90 ± 0.23 minutes) than with tramadol (4.61 ± 0.38 minutes) (*P* < .001).

Megalla and Mansour^6^ compared nalbuphine, dexmedetomidine, and saline and reported that the meantime to control shivering in the dexmedetomidine group was 1.97 ± 0.61 minutes which was considerably lower than the other 2 groups. The time to control shivering was significantly less in the dexmedetomidine group when compared with the nalbuphine group (*P*  = .04) and with the control saline group (*P*  = .0001). The shivering control time was also significant when the nalbuphine group was compared with the control saline group (*P*  = .0001).

Mohamad^11^ compared the dexmedetomidine 0.5 μg kg^−1^ (Group D) and Nefopam 0.15 mg kg^−1^ (Group N) in 50 patients per group. The mean time to control shivering in the dexmedetomidine group was 4.63 ± 1.19 minutes which was significantly higher than the nefopam group (2.35 ± 0.67 minutes) (*P*  = .031).

Panneer et al^12^ compared intravenous clonidine to dexmedetomidine group and reported that dexmedetomidine took 2.23 ± 0.43 minutes to control shivering compared to clonidine with 5.54 ± 0.58 minutes (*P* < .01).

Venkatraman et al^13^ compared 3 groups in patients with Group T receiving tramadol, Group C receiving clonidine, and Group D receiving dexmedetomidine and concluded that the mean time to resolve shivering with dexmedetomidine was 5.76 ± 1.14 minutes which was significantly less than tramadol (6.72 ± 1.27 minutes) and clonidine (9.48 ± 0.95 minutes), (*P* < .0001) using one-way ANOVA test.

Bansal and Jain^10^ reported a comparison of tramadol 50 mg intravenous bolus, butorphanol 1 mg intravenous bolus, and clonidine 150 μg intravenous bolus in 30 patients per group. The time taken for control of shivering was significantly higher (3.3 ± 0.9 minutes) in the clonidine group than in the tramadol group (2.1 ± 1.0 minutes) and butorphanol group (1.8 ± 0.5 minutes) (*P* < .001), though the difference between tramadol and butorphanol was insignificant (*P* = .13).

Chan et al^4^ compared 2 doses of intravenous tramadol (T0.5 = 0.5 mg kg^−1^ and T0.25 = 0.25 mg kg^−1^) with saline and reported that although shivering control was better with a dose of 0.25 mg kg^−1^ tramadol, shivering cessation earlier with a dose of 0.5 mg kg^−1^ of tramadol. The time to control shivering was shorter in group T0.5 (3.8 minutes, 95% CI 2.3-5.3 minutes) and group T0.25 (5.4 minutes, 95% CI 3.7-7.1 minutes) as compared to group saline (10 minutes, 95% CI 0.83-20.8 minutes) (*P* < .01).

Kaya et al^7^ compared meperidine 0.35 mg kg^−1^ (n = 30) with tramadol 0.25 mg kg^−1^ (n = 30) and stated that tramadol took 155 ± 64 seconds to stop shivering as compared to the meperidine 181 ± 89 seconds. The time to control shivering by tramadol was statistically insignificant when compared with meperidine (*P*  = .19) using two-sample *t*-test in STATA software.

Bansal and Jain^10^ reported that butorphanol takes less time to resolve shivering than tramadol and clonidine. The time taken for the complete cessation of shivering was significantly higher (3.3 ± 0.9 minutes) in the clonidine group than in the tramadol group (2.1 ± 1.0 minutes) and butorphanol group (1.8 ± 0.5 minutes) (*P* < .001), though the difference between tramadol and butorphanol was insignificant (*P * = .13).

### Recurrence of Shivering

Five studies reported recurrence of shivering out of 10.^6,7,9,10,12^

#### Studies with Dexmedetomidine Treatment

For dexmedetomidine, 4 studies mentioned the recurrence rate.^6,9,10,12^ In these studies, a total of 135 patients received dexmedetomidine in a dose of 0.5 µg kg^−1^ and shivering recurred in 1 patient only. The total rate of recurrence of shivering with dexmedetomidine was 0.74%.

#### Studies with Tramadol Treatment

Out of 6 studies, tramadol was used in 3 studies in different doses.^7,9,10^ In these studies, a total of 110 patients received tramadol at different doses 0.25 mg kg^−1^,^7^ 0.5 mg kg^−1^,^10^ and 1 mg kg^−1^.^9^ Shivering recurred in 17 patients. The cumulative recurrence rate of shivering with tramadol (0.25-1 mg kg^−1^) was 15.45%.

#### Studies with Butorphanol Treatment

Butorphanol was used in 1 study,^13^ which mentioned the recurrence rate. A total of 30 patients received it and 2 patients developed a recurrence of shivering. The rate of recurrence of shivering with butorphanol was 6.7%.

#### Studies with Clonidine Treatment

For clonidine, 2 studies mentioned the recurrence rate.^9,12^ In these studies, a total of 60 patients received clonidine in the dose of 1 µg kg^−1^ and shivering recurred in 11 patients. The cumulative rate of recurrence of shivering with clonidine is 18.3%.

#### Studies with Meperidine Treatment

Meperidine was used in different doses in a study by Kaya et al^7^ which mentions the recurrence rate. A total of 30 patients received it and none of them developed a recurrence of shivering. The rate of recurrence of shivering with butorphanol was 0%.

The rest of the other drugs were used in single studies in a small number of patients and the rate of recurrence based on single studies cannot be commented on.

### Adverse Effects

Opioid-related side effects such as nausea and vomiting were reported in almost all included studies. Additionally, hypotension was observed in 6 studies,^5,6,8,11,12^ bradycardia was observed in 7 studies,^5,6,8,9,10-12^ and sedation was reported in 8 studies.^6-13^

#### Adverse Effects with Dexmedetomidine and Tramadol

Fern and Misiran^8^ compared the effectiveness of intravenous dexmedetomidine with that of pethidine and tramadol. The sedation levels were similar among all 3 groups, but the higher frequency of hypotension and bradycardia was witnessed in the dexmedetomidine group (*P* < .05). A single patient in the tramadol group reported nausea and vomiting symptoms.

Kundra et al^9^ studied the effect of intravenous dexmedetomidine with tramadol in patients for control of shivering. In the tramadol group, nausea and vomiting were reported as adverse events compared to no case in the dexmedetomidine group. Although moderate sedation and bradycardia were witnessed in the dexmedetomidine group, it provided better results than the tramadol group.

Megalla and Mansour^6^ compared nalbuphine, dexmedetomidine, and saline and observed that among the adverse events, sedation was observed in both groups of nalbuphine and dexmedetomidine. In the dexmedetomidine group, bradycardia and hypotension were more common.

Venkatraman et al^13^ compared tramadol, clonidine, and dexmedetomidine for control of intraoperative shivering. Dexmedetomidine caused more significant sedation as compared to the other 2 groups. Hypotension was observed more in the dexmedetomidine group, whereas most of the vomiting cases were reported in the tramadol group.

Kaya et al^7^ compared meperidine with tramadol and found that 2 patients from tramadol and 4 patients from the meperidine group had drug-related nausea, but none developed vomiting. No difference in the sedation score was noticed between the 2 groups.

#### Adverse Effects with Butorphanol

Bansal and Jain^10^ reported that adverse events such as nausea and vomiting were observed less in the group in which butorphanol was used as compared to group tramadol and clonidine. However, a higher incidence of grades 1 and 2 sedation was noted in the butorphanol group compared with other groups.

#### Adverse Effects with Other Medications

Ibrahim et al^5^ compared saline, prophylactic dose, and therapeutic dose of magnesium sulfate. Nausea and vomiting were reported more in magnesium sulfate groups than the control group, whereas hypotension was more common in the prophylactic group.

Mohamed^11^ compared the effectiveness of intravenous dexmedetomidine and nefopam to treat and control intraoperative shivering. Adverse effects like bradycardia, hypotension, and sedation were observed only in the dexmedetomidine group.

#### Comparable Adverse Effects

Chan et al^4^ compared 2 doses of intravenous tramadol and found no difference in the response rate and the side effects between the 2 doses. Similarly, not a single patient in any group developed sedation or desaturation.

Panneer et al^12^ compared clonidine and dexmedetomidine and concluded that the frequency of nausea and vomiting were similar in both groups, while better sedation level and stable cardiorespiratory parameters were observed in the dexmedetomidine group.

### Meta-Analysis

Meta-analyses were done for the first 2 outcomes, that is, treatment and control of shivering and time to control shivering.

**Figure 1.**
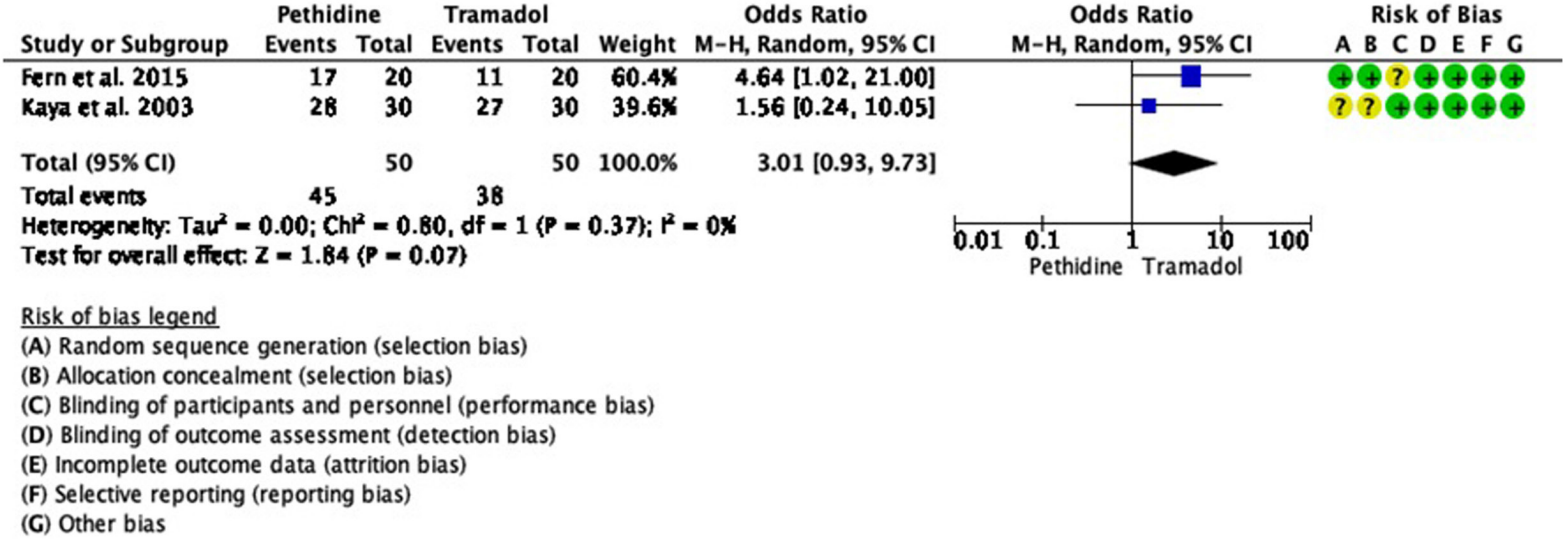
Forest plot for control of shivering (Tramadol versus Pethidine).

Data from the included studies were analyzed. We selected studies that used intravenous tramadol, dexmedetomidine, and pethidine with similar comparison for 2 specific outcomes. We analyzed the effectiveness of tramadol versus pethidine for the control of intraoperative shivering from 2 studies.^7,8^ The overall OR was 3.01 [95% CI, 0.93-9.73]. The results were more toward the favor of tramadol, but the interval estimates of individual studies and overall prism were statistically insignificant (Figure 1).

**Figure 2.**
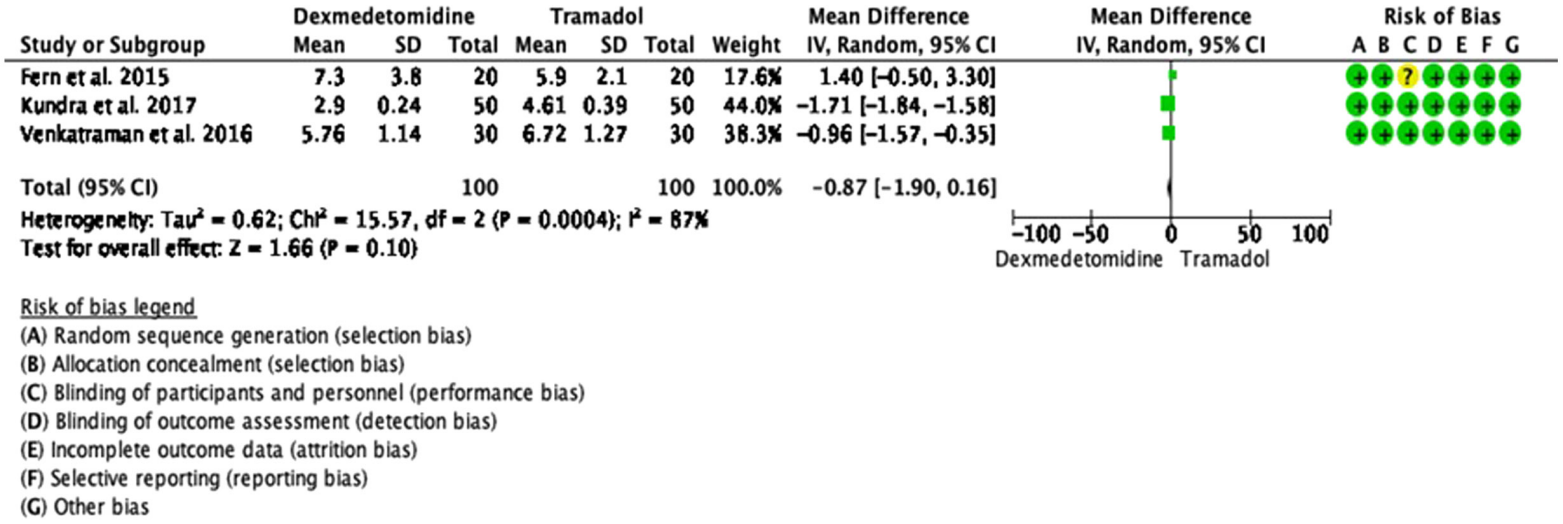
Forest plot for time to control shivering (Dexmedetomidine versus Tramadol).

The mean time taken to stop shivering was also analyzed from studies with similar outcome comparisons.^8-10 [Bibr b11-tjar-50-4-246]^ Dexmedetomidine and tramadol were comparable (MD −0.87 [95% CI −1.90-0.16]) and statistically insignificant (Figure 2).

## Discussion

In this systematic review, we found that opioids, centrally acting α2 receptor agonist, magnesium sulfate, amitriptyline, and nefopam were used from most to least, respectively, in the included trials. Among different opioids, tramadol was the most common drug, followed by pethidine/meperidine in the trials we have reported. Among α2 receptor agonists, dexmedetomidine and clonidine were used.

Intravenous tramadol was used in different doses, ranging from 0.25 mg kg^−1^ to 1 mg kg^−1^. Intravenous tramadol was used in the doses of 0.25 mg kg^−1^ in 2 studies,^4,7^ 0.5 mg kg^−1^ in 3 studies,^4,8,10^ and 1 mg kg^−1^ in 1 study.^9^ It was also used as a 50 mg intravenous bolus in 1 study by Bansal and Jain.^10^ The most commonly used intravenous tramadol dose for treating post spinal shivering was 0.5 mg kg^−1^. The intravenous dose of pethidine/meperidine was ranging from 0.35 mg kg^−1^ to 0.5 mg kg^−1^ or as a 50 mg intravenous bolus. Intravenous pethidine was used in doses of 0.35 mg kg^−1^ in 1 study^7^ and 0.5 mg kg^−1^ in another study.^8^ Intravenous nalbuphine (0.07 mg kg^−1^) was also used in 1 study to control shivering.^6^ Intravenous butorphanol has also been used as an intravenous bolus of 1 mg in a single study.^13^

In almost all of the included studies^5,6,8,10-12^ with dexmedetomidine, a dose of 0.5 μg kg^−1^ has been used. Intravenous clonidine was used in the dose of 1 μg kg^−1^ in 2 studies^9,12^ and as an intravenous bolus of 150 μg kg^−1^ in 1 study.^13^

**Figure 3.**
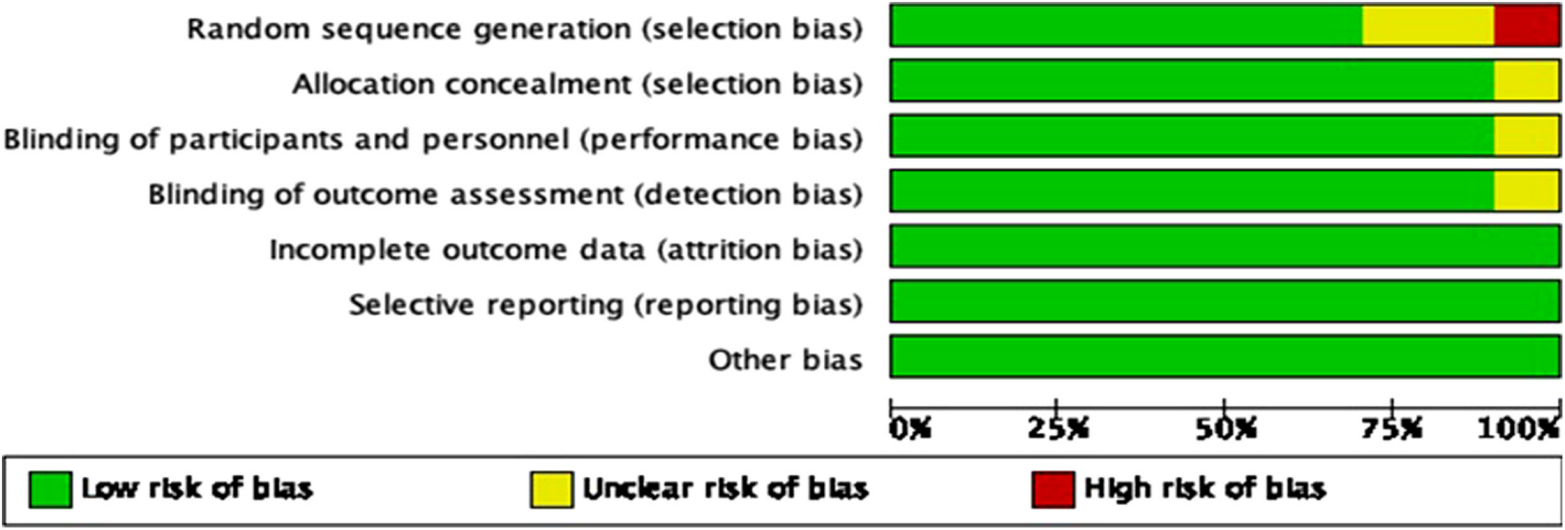
Percentage of risk of bias.

**Figure 4.**
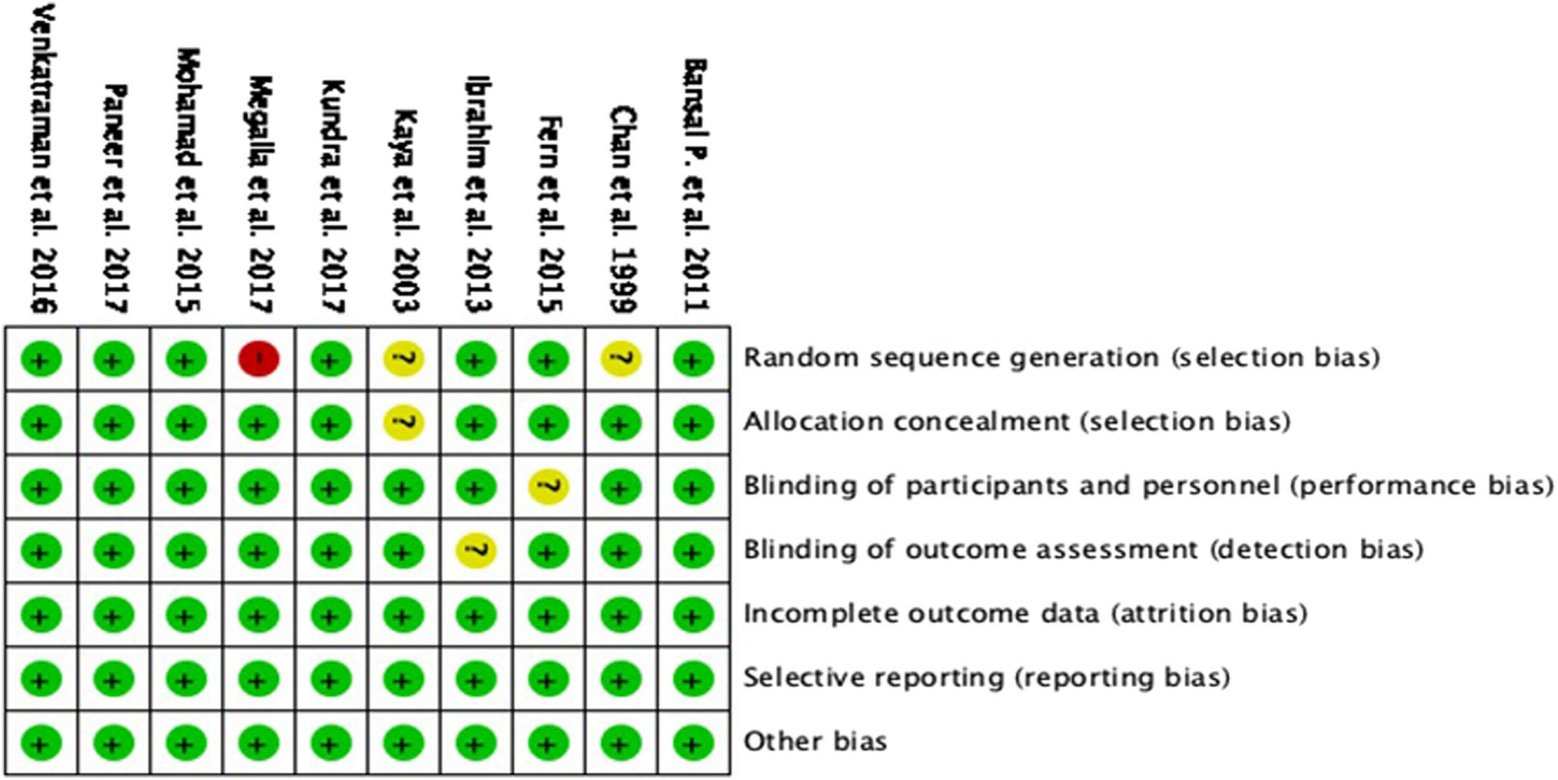
Risk of bias for included trials.

Each included study was also assessed for quality. Common biases of the RCT were examined independently by each reviewer and consensus was made. Most of the included studies were of good quality (Figures 3 and 4). Studies with a high risk of selection, performance, and detection bias and/or other biases were excluded from results and analysis. Brief details of some of the excluded studies are given below.

In one excluded study by Keerthi and Kamath^15^ details of randomization were not mentioned. It was only mentioned that “patients were randomly distributed into 3 groups of 32 patients each.” Performance and detection bias was also at high risk. In Table 2 of that study, the means and standard deviations of heart rate (HR) for the butorphanol and tramadol groups were identical down to the 60-minute point. The same thing can be seen in the diastolic blood pressure (BP) graph in Supplementary Figure 1 of that study. In the study by Mahesh and Kaparti^16^ detection and selective reporting bias were observed. It was not mentioned whether the attending anaesthesiologist was there for outcome assessment and also it only reported significant findings of the study. Another study by Abdel-Ghaffar et al^14^ was also excluded because, in the weights of subjects in the Dex II and Dex III groups, the mean in each case falls outside of the stated ranges, which is impossible. Few other studies were also excluded due to various quality-related issues and biases.

If we discuss the results of included studies by specified outcomes, it showed that dexmedetomidine and tramadol were the 2 most commonly used drugs with their effect on the control of shivering (or response rate) after central neuraxial block. The average time from the administration of drugs to the complete disappearance of shivering was also slightly less with dexmedetomidine (i.e., 4.13 ± 1.23 minutes) than that of tramadol (i.e., 4.55 ± 1.16 minutes), but it seems clinically insignificant. If we see the recurrence of shivering as a categorical outcome and compare dexmedetomidine and tramadol from the included studies, the recurrence of shivering is found to be more frequent when tramadol was used (i.e., 15.45%); however, it was near to no recurrence with dexmedetomidine use (i.e., 0.74%). With the finding listed above, dexmedetomidine seems more effective with a relatively shorter onset of action and a negligible chance of recurrence of shivering. Haemodynamic adverse effects (bradycardia and hypotension) were more significant with the use of dexmedetomidine, while nausea and vomiting were more significant with the tramadol use. However, sedation was similar in both drugs.

Among the 10 included studies, only 2 studies were selected to analyze the effect of tramadol versus pethidine for the control of shivering (Figure 1). It showed a statistically insignificant result while the heterogeneity in the studies was very low, with a value of I^2^ = 0%. By observing the overall effect of interventions, the confidence intervals were more skewed toward the favor of tramadol than pethidine, but the cumulative effect was statistically insignificant. About 3 studies were included in determining the effect of dexmedetomidine versus tramadol for the 2nd listed outcome of time to control shivering (Figure 2). It showed that there was no difference in the time to control shivering between tramadol and dexmedetomidine. The main limitation of this meta-analysis was the high heterogeneity level (i.e., the value of I^2^ = 87%). The MD of time to control shivering was statistically insignificant −0.87 [95% CI −1.90-0.16]. The possible causes of heterogeneity in the meta-analysis for the time to control shivering was the difference in population characteristics and doses of interventions. The lower limit of age in the population of included studies was almost similar (i.e., >18 years) with some difference in the upper age limit like it was 70 years in Fern and Misiran^8^ and Venkatraman et al^9, 13^ and no upper limit was mentioned in Kundra et al.^9^ In addition to the difference in population age range, the doses of tramadol were also different. Intravenous tramadol was administered in the dose of 0.5 mg kg^−1^ in 3 studies,^8,10^ while it was 1 mg kg^−1^ in 1 study by Venkatraman et al.^13^

There exists a gap in the primary literature due to the lack of good quality large RCT. We summarized our results and meta-analysis based on relatively small existing clinical trials and because of limited number of studies meta-analysis may be of limited benefit. Finding the most cost-effective intervention(s) with a standard dose and regimen should be addressed in future studies. Working for non-pharmacological interventions like acupressure and different warming techniques should also be considered for future studies.

## Conclusion

In conclusion, the available data regarding the pharmacological agents used to treat shivering after central neuraxial anaesthesia were presynaptic α2 receptor agonists including dexmedetomidine and clonidine and opioids including tramadol, pethidine, nalbuphine, and butorphanol (Supplementary Tables 3 and 4). The most effective drug with approximately 100% response rate was dexmedetomidine with the dose of 0.5 μg kg^−1^ intravenously given just after the appearance of shivering. Clonidine has also been used successfully with some side effects. The common side effects of α2 receptor agonists were hypotension and bradycardia. Studies also showed that opioids, like tramadol, is also an effective drug used to control shivering in most of the patients and this effect is comparable with that of pethidine (Figure 1). Nausea and vomiting were the main adverse effects when opioids were used. The other successful opioid intervention in the included trials apart from tramadol and pethidine was butorphanol.

## Figures and Tables

**Supplementary Table 1.** Characteristics of Included Studies

**S. No.**	**Study**	**Journal**	**Decision**	**Reasons/Comments**
1.	Chan et al^4^ 1999	Canadian Journal of Anesthesia	Included	- No apparent discrepancy seen in result tables - Random sequence generation unclear only
2.	Ibrahim et al^5^ 2013	Egyptian Journal of Anaesthesia	Included	- No apparent discrepancy seen in result tables
3.	Megalla and Mansour^6^ 2017	Egyptian Journal of Anaesthesia	Included	- No apparent discrepancy in result seen
4.	Kaya et al^7^ 2003	European Journal of Anaesthesiology	Included	- No major quality issue - No result discrepancies
5.	Fern and Misiran^8^ 2015	Southern African Journal of Anaesthesia and Analgesia	Included	- No apparent discrepancy seen in result tables- Recently included in meta-analysis
6.	Kundra et al^l9^ 2017	Saudi Journal of Anaesthesia	Included	- No apparent discrepancy in result seen - Study included in other published meta-analysis
7.	Bansal and Jain^10^ 2011	Local and regional anaesthesia	Included	- Risk of selection bias unclear only - No apparent discrepancy seen in result tables
8.	Mohamed^11^ 2015	Egyptian Journal of Anaesthesia	Included	- No apparent discrepancy in result seen
9.	Panneer et al^12^ 2017	Anaesthesia, Essays, and Researches	Included	- No apparent discrepancy in result seen
10.	Venkatraman et al^13^ 2016	Revista brasileira de anestesiologia	Included	- No apparent discrepancy in result seen - Study used in other meta-analysis also - Only time to control shivering and recurrence was observed - Response to stop shivering was not observed

**Supplementary Table 2.** Characteristics of Excluded Studies

**S. No.**	**Study**	**Journal**	**Decision**	**Reasons/Comments**
1.	Abdel-Ghaffar^14^ 2015	*Pain Physician*	Excluded	In weights of the subjects in the Dex. II and Dex. III groups, mean in each case falls outside of the stated ranges, which is impossible
2.	Keerthi and Kamath^15^ 2017	*Research Journal of Pharmaceutical, Biological and Chemical Sciences*	Excluded	- In Table 2, the means and standard deviations of HR for the butorphanol and tramadol groups are identical all the way down to the 60-minute point - The same thing can be seen in the graph of the diastolic BP (Figure 1)
3.	Mahesh and Kaparti^16^ 2014	*Journal of Clinical and Diagnostic Research*	Excluded	- Suspicious results see Tables 4 and 7 - High risk of selection bias, performance bias, and detection bias
4.	Shukla et al^17^ 2011	*Indian Journal of Anaesthesia*	Excluded	- Poor quality - No apparent discrepancy in results seen - Study included in other published meta-analysis for clonidine
5.	Manne and Gondi^18^ 2017	*Anaesthesia, Essays, and Researches*	Excluded	- Suspicious results, mistakes in Table 1. - High risk/unclear selection bias, performance bias, detection bias, attrition bias, and reporting bias
6.	Palan et al^19^ 2017	*Journal of Krishna Institute of Medical Sciences University*	Excluded	- Suspicious results. Shivering resolved in majority within 60 seconds - Selection bias, performance bias, and detection bias
7.	Casey et al^20^ 1988	*Canadian Journal of Anaesthesia*	Excluded	- High risk of selection bias, performance bias, performance bias, and detection bias - No apparent discrepancy seen in result tables
8.	Tsai and Chu^21^ 2001	*Anaesthesia and Analgesia*	Excluded	- Poor quality study with different biases - No apparent discrepancy in result seen
9.	Joshi et al^22^ 2013	*Anaesth Pain Intensive Care*	Excluded	- Poor quality study with different biases - Cited only once since 2013 - No apparent discrepancy in result seen
10.	Brownridge^23^ 1986	*Anaesthesia and Intensive Care*	Excluded	- Patient did not undergo surgery
11.	Capogna and Celleno^24^ 1993	*British Journal of Anaesthesia*	Excluded	- No surgery was planned - Observed shivering after delivery which does not match defined objective - NVD v/s LSCS (crossover)
12.	Chen et al^25^ 1994	*The Kaohsiung Journal of Medical Sciences*	Excluded	- Non-English publication
13.	Harris et al^26^ 1989	*Regional Anaesthesia*	Excluded	- Excluded as intervention drug was given before regional anaesthesia
14.	Juneja et al^27^ 1992	*Journal of Clinical Anaesthesia*	Excluded	- Excluded as patient did not undergo surgery
15.	Koay et al^28^ 1991	*Singapore Med. Journal*	Excluded	- It is not an RCT. It is a quasi-experimental study
16.	Mercadante et al^29^ 1994	*Journal of Pain and Symptom Management*	Excluded	- Patient did not undergo surgery

RCT, randomized controlled trials.

**Supplementary Table 3.** Summary Table for Control of Shivering

	Variables	Dexmedetomidine	Clonidine	Tramadol	Butorphanol	Pethidine	Nalbuphine	Nefopam	Placebo/Saline
Fern and Misiran	Response rate	100%	NA	55%	NA	85%	NA	NA	NA
Megalla and Mansour	Success rate	100%	NA	NA	NA	NA	92%	NA	32%
Mohamed	Response rate	96%	NA	NA	NA	NA	NA	100%	NA
Paneer et al	Response rate	100%	80%	NA	NA	NA	NA	NA	NA
Kundra et al	Shivering disappearance	100%	NA	100%	NA	NA	NA	NA	NA
Bansal and Jain	Shivering control rate	NA	53.3%	73.3%	83.3%	NA	NA	NA	NA
Kaya et al	Response rate	NA	NA	90%	NA	93%	NA	NA	NA

Green shade represent most effective; yellow shade represent intermediate effect; Red shade represent least effective; NA, not applicable.

**Supplementary Table 4.** Summary Table for Time to Control Shivering

	Dexmedetomidine	Clonidine	Tramadol	Butorphanol	Pethidine	Nalbuphine	Nefopam	Placebo/Saline
Fern and Misiran	7.3 ± 3.8 minutes	NA	5.9 ± 2.1 minutes	NA	6.2 ± 2.3 minutes	NA	NA	NA
Bansal and Jain	NA	3.3 ± 0.9 minutes	2.1 ± 1.0 minutes	1.8 ± 0.5 minutes	NA	NA	NA	NA
Megalla and Mansour	1.97 ± 0.61 minutes	NA	NA	NA	NA	3.56 ± 0.82 minutes	NA	12.4 ± 3.74
Mohamed	4.63 ± 1.19 minutes	NA	NA	NA	NA	NA	2.35 ± 0.67 minutes	NA
Paneer et al	2.23 ± 0.43 minutes	5.54 ± 0.58 minutes	NA	NA	NA	NA	NA	NA
Kundra et al	2.9 ± 0.23 minutes	NA	4.61 ± 0.38 minutes	NA	NA	NA	NA	NA
Venkatraman et al	5.76 ± 1.14 minutes	9.48 ± 0.95 minutes	6.72 ± 1.27 minutes	NA	NA	NA	NA	NA
Kaya et al	NA	NA	155 ± 64 seconds	NA	181 ± 89 seconds	NA	NA	NA

Green shade represent minimum; yellow shade represent intermediate; Red shade represent maximum; NA, not applicable.
